# Study on Smart Home Interface Design Characteristics Considering the Influence of Age Difference: Focusing on Sliders

**DOI:** 10.3389/fpsyg.2022.828545

**Published:** 2022-03-22

**Authors:** Na Yu, Ziwei Ouyang, Hehe Wang

**Affiliations:** ^1^College of Furnishings and Industrial Design, Nanjing Forestry University, Nanjing, China; ^2^Co-Innovation Center of Efficient Processing and Utilization of Forest Resources, Nanjing Forestry University, Nanjing, China; ^3^College of Art and Design, Nanjing Forestry University, Nanjing, China

**Keywords:** smart home, user interface, age different, sliders, design characteristics

## Abstract

Smart homes represent an effective approach to improve one’s quality of life. Developing user interfaces that are both comfortable and understandable can assist users, particularly the elderly, embrace smart home technologies. It’s critical to concentrate on the characteristics of smart home interface design and their impact on people of various ages. Since sliders are one of the most common components utilized in the smart home user interface, this article aimed to investigate the effects of slider design characteristics (e.g., button size, track color, and sliding orientation) on user performance and preference. Thirty-four participants were recruited for the experiment (16 for the young group, aged between 18 and 44 years; 18 for the middle-aged and elderly group, aged between 45 years and above). Our results revealed that both groups had shorter task completion time, less fixation time, and saccades on horizontal sliding orientation and larger buttons, which means better user performance. For the older group, the slider with color gradient track led to better user performance, while the track color only had less effect on the performance of the younger group. In terms of user preference, the results and performance of the older group were basically consistent, while the younger group had no significant difference in sliding orientation and track color.

## Highlights

- Different age groups showed the same performance for sliding orientation factors, while the results of track color and button size were different.
- The youth group performed better in horizontal sliding direction and button size of 5 mm and above, with a preference for button size of 5 mm, while track color only had less influence on user performance.
- The middle-aged and elderly group performed better in horizontal sliding direction, color gradient track, and button size of 5 mm and above.

## Introduction

Ambient assistive living (AAL) is a technological approach to help individuals in everyday activities (including work) in spite of environmental challenges or personal physical and cognitive impairments. When this form of technology is applied to the aging population, it improves the elderly’s quality of life and reduces caregivers’ burdens (Panico et al., 2020). Smart homes represent a promising approach to providing AAL (Hwang and Hoey, 2012).

Smart homes are the application of Internet of things (IoT) technology in the home environment. It plays an important role in providing users with a convenient, efficient, and high-quality life (Gunge and Yalagi, 2016). A smart home can control and monitor the environmental variables of someone’s home (Liao et al., 2019), identify user activities, detect anomalies (Fahad and Tahir, 2020), and help households to better control home equipment (Bissoli et al., 2019). It also shows a positive relationship between the use of smart home services and wellbeing (Sequeiros et al., 2021). The benefits of smart home technology have been confirmed by many studies, but the existing study predominantly focuses on the technical characteristics of a smart home, which means there is a lack of user perspective, especially in eastern countries (Marikyan et al., 2019).

As a comfortable and understandable user interface can help to develop a user-centric smart home, it is of great significance to study the interface design characteristics of a smart home (Borodulkin et al., 2002). Research has been carried out around the key layout form (Sharma and Wong, 2020), information grouping method (Jeong et al., 2012), and intelligence level (Zhang et al., 2009). However, interacting with a smart home still poses challenges, especially for the elderly. The senior users assessed in a study on their views and expectations about smart home technology have a generally positive attitude toward smart technology, but there is also concern about the use of technology that is affecting their use decisions (Mann et al., 2007). Users are more hesitant to adopt smart home technology when they have a high level of technology anxiety (Holden and Karsh, 2010). When it comes to interacting with smart homes, research shows that there are distinctions between young and senior users (Zhang et al., 2009). With the increasingly serious global aging problem, it is vital to consider the impact of age differences on user performance and preferences when conducting research on the smart home interface.

Through our preliminary market research, we found that the slider is one of the basic components of the smart home terminal interface, and it is mainly used for the number of types of feature sets used, such as time adjustment, temperature control, humidity control, fresh air system for wind speed adjustment, light RGB value adjustment, lighting brightness adjustment, the curtain open degree adjustment, and background music volume adjustment functions.

The purpose of this study was, therefore, to take sliders as the experimental object and reveal how design characteristics affect users of different ages, so as to provide a reference for designing smart home interfaces that are optimized for diverse users.

## Related Work

### Touchscreen Interaction

Touchscreen technologies have become increasingly common in personal devices because of their natural and convenient human-machine interaction (Tao et al., 2018). The use of touchscreen technology has many advantages. For example, compared with input devices such as mouse and keyboard, a touchscreen can be easily operated by inexperienced users, which greatly improves user operability (Ahearne et al., 2015). Although, when using touch technology, the performance of an age-related difference is small, making the technology fully accommodate the requirements of users of different ages still needs the effort. As designers, we should focus on the characteristics of users including perceptual, psychomotor, cognitive, and physical changes. Understanding the different age capabilities and limitations can help to create higher usability interface (Niamh et al., 2012). Finding a personalized design approach based on individual preferences can empower the users and mitigate erroneous representations, especially when the elderly are represented by a highly heterogeneous group (Menghi et al., 2017). The development of accessible, ergonomic, and user-friendly interfaces can enable older people to benefit from touchscreen devices, prevent digital exclusion, and improve the quality of life (Lilian et al., 2014).

### Slider Component

Sliders are widely used in user interfaces for touchscreens. The interaction approach it offers is press-drag, which means the user presses the slider component at the thumb and drags it to the desired release point, mostly for stepless adjustment. But as highlighted in Nielsen Norman Group, sliders are difficult to manipulate. Both the visual style and orientation of the slider will affect the precision of the entered value (Colley et al., 2019). Since the area where the finger first touches the slider is covered by the finger itself, the button size of the slider is also crucial to the availability of the slider component. Therefore, our experiment selected button size, track color, and sliding orientation as the design characteristics to be studied.

### Button Size

Button size is an important factor in interface interaction. Its influence covers the aspects of interaction performance, user mental load, and preference. There are standards for the size of buttons. American standard (ANSI/HFES 100, 2007) recommends a minimum key size of 9.5 mm (ANSI/HFES 100, 2007), while in ISO 9241-9, the recommended button size can be the breadth of the male distal finger joint at the 95th percentile, which is approximately 22–23 mm (Standards, 2000). However, the research results of relevant scholars deviate from the recommended size of the previous standard. The optimal size of the buttons obtained by them through experiments is 19.05 mm (Zhao et al., 2007) and 20 mm (Colle and Hiszem, 2004; Chen et al., 2013), which is closer to the ISO standard. Previous studies have shown that larger button sizes lead to better interaction, and this result was reflected in different task types. For example, users perform better when the button size is large (i.e., 17.5 mm and above) in the task of digit and letter input (Tao et al., 2018). In game tasks, whether physical solid or touch buttons, users performed better using 1.1 cm^2^ keys than 0.6 cm^2^ keys (Lin et al., 2019). In the virtual reality environment, the button size of 15 mm will be unavailable. With the increase in the button size, the task completion time and the error time will decrease, and the optimal button size is 25 mm (Park et al., 2020). In the case of one-handed thumb operation of mobile handheld devices, the optimal button size obtained varies. The study of Ouyang XW concluded that increasing the size of the button on a smartphone from 8 to 14 mm can improve the task completion rate and the task efficiency in the screen mirror of older adults when they click with one-handed posture (Ouyang et al., 2021). The results of Parhi showed that the task completion time of the subjects decreased with the increase in the target size, but when the target was larger than a certain size, there was no significant difference in the click operation error rate among different sizes, among which the button size was 9.6 mm for discrete click operation and 7.7 mm for continuous click operation (Parhi et al., 2006). Yong further concluded that the completion time of single-hand operation with 7 and 10 mm key size is the shortest, while the operation error is the least and subjective satisfaction is the highest with 10 mm button size (Yong and Han, 2010). Not only in terms of the interaction effect, the size of the button will also affect the physical health of users by affecting forces, impulses, and dwell times for participants completing tasks on a touch screen (Sesto et al., 2012). However, button function (Park et al., 2020), user posture (Amrish et al., 2013), and screen size (Hancock et al., 2015) all affect the users’ demand for button size, and existing studies are unable to provide a reference for the button size design based on the tablet size in a smart home environment. Moreover, most of the existing research targets are press buttons, and there is a lack of research on sliding buttons. In the smart home system, most of the adjustment buttons are in the nonpolar adjustment mode, that is, the sliding buttons are the main.

### Color

As a feature, the color will capture attention as a distractor (Snowden, 2002) or guide attention as a target (Biggs et al., 2015). In the design of smart home slider interactive buttons, it is worth exploring whether the color of the slider components can guide users to interact efficiently, and under what conditions the effect is the best. Color will affect the users’ cognitive performance (Bhattacharyya et al., 2014), and the individual colors differed significantly in their level of guidance of attention (Andersen and Maier, 2019). In addition, the combination of the color of the readability of the information display system also has a great influence (Humar et al., 2008). In the design of data visualization, two types of phenomena should be considered, namely, simultaneous color contrast (Mittelstädt et al., 2014) and successive contrast (Geisler, 1978). That is, the interaction between adjacent or sequentially displayed colors will lead to perceptual bias. When the legibility of information is low, the users’ reading time will increase (Naujoks et al., 2019). Existing studies on interface color include the color contrast between buttons and text (Jung et al., 2021) and the influence of interface background color on user emotion (Cheng et al., 2019). As for the color study of buttons, Huang found that the color combination of the graphics and background in the button icon affected the visual search performance. The higher the color contrast, the better the user search performance of the subject (Huang, 2008). Sha confirmed this conclusion in elderly subjects (Sha et al., 2017). However, these studies are all based on touch screen press buttons, and there is still a gap in the study of the track color of slider components.

### Orientation

For sliding components, the interaction modes mainly include horizontal sliding, vertical sliding, and annular sliding. Poor sliding component design will lead to problems such as mismatch between input results and user intentions, resulting in low user experience. Colley studied the impact of visual style and sliding orientation of touch screen slider on input accuracy and compared the difference between horizontal and vertical sliding (Colley et al., 2019). But most of the research on sliders is on non-touch physical sliders. For example, Scott reported that the input value in the horizontal direction was slightly lower than that in the vertical direction (Scott and Huskisson, 1979), and Paul studied the influence of the end point and direction effect of sliding components (Paul-Dauphin et al., 1999). Which means there are few studies on the sliding components of touch screens.

### Study Hypotheses

In order to explore the influence mechanism of different forms of sliding buttons in the smart home interface on user performance of different age groups, this study carried out experimental research from sliding direction, sliding track color, and slider button size to explore the optimal recognition efficiency and user preference, so as to optimize the design.

**Hypothesis 1:** The larger the button size, the better the task performance.

**Hypothesis 2:** Slide track color has an indicative role for the user.

**Hypothesis 3:** Users will prefer horizontal swiping interaction.

Hypothesis 1 was based on the common findings of previous scholars. Hypothesis 2 was proposed according to our expectations on the color that can guide attention. Hypothesis 3 was proposed based on Colley’s previous research results (Colley et al., 2019).

## Materials and Methods

### Experimental Subjects Selection

Through network research and field research, the sliding orientation, track color, and button size were analyzed to provide a reference for experimental design. The research objects mainly include some well-known brands in the Chinese smart home market, such as Midea Merlot, Haier Smart Home, Gree++, TCL Smart Home, Xiaomi Mijia, Huawei HiLink, Hedong, UIOT, Honeywell, Orvibo, and LivingLab; Moorgen of Germany; and Savant of the United States, which target high-spending users.

**FIGURE 1:**
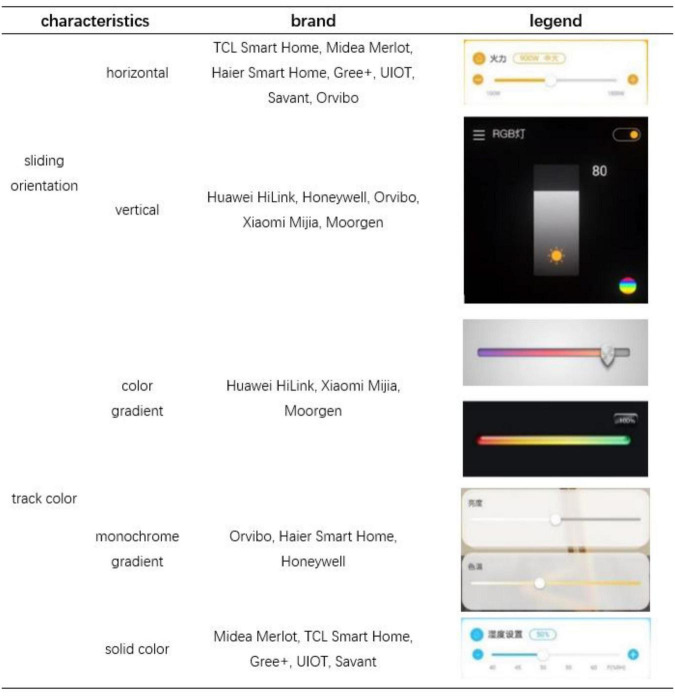
Smart home sliding component survey. The slider orientation in the Chinese smart home market is mainly horizontal and vertical, and the track color can be divided into color gradient, monochrome gradient, and solid color.

Through investigation and analysis of offline stores, online websites, and application software of smart home brands, the features of sliding buttons are summarized as shown in Figure 1. For the sliding buttons of the smart home, the display characteristics of various brands are not the same. Many of them are set to slide in the horizontal orientation, and some of them are vertical orientation. The interface of Orvibo has both horizontal orientation and vertical orientation sliding buttons. In terms of track color, most brands have solid color slider interfaces, and a few have monochrome or color gradient forms. The plain color interface is more concise and clear, but the gradient color may have a certain cue effect, which will be further discussed in the subsequent experiments. According to the preliminary investigation, we chose temperature setting as the case study and determined the experimental factors.

### Experimental Design

This study implemented a three-factor (3*5*2) within-subject design, with button size, track color, and sliding orientation serving as independent variables. According to the preliminary investigation, the experimental factors are determined. The button size is the diameter of the circular slider. Through investigation and measurement, it is concluded that the size of the button on the intelligent terminal interface ranges from 3 to 7 mm. Three levels are selected for equal division: 3, 5, and 7 mm. The sliding orientation is the gesture direction of dragging the sliding control slider, and two levels are selected, namely, horizontal and vertical. The track color is the progress bar color of the track where the slider is located. Five levels are selected, namely, color gradient, blue gradient, blue solid color, gray gradient, and gray solid color.

User performance was measured by objective evaluation indexes (i.e., task completion time and eye movement data including saccade times and mean fixation time), and user preference was measured by subjective evaluation index (i.e., user preference questionnaire). Task completion time referred to the total time spent by a participant to complete a task. Eye movements were sampled using an eye tracker. Saccade times and mean fixation time were used to measure the searching efficiency and cognitive load of the participants during the tasks. User preference was assessed through a paper questionnaire that was used to investigate their most preferred button design. In the questionnaire, schematics and descriptions of the three factors at all levels with equal proportion were listed (see Appendix A), from which the participants were required to select their favorite button design for smart home adjustments.

### Participants

Through open recruitment on campus, a total of 34 Chinese people participated in the study. The subjects were divided into two groups according to age, namely, youth group (aged 18–45 years, including 18 years but excluding 45 years) and middle-aged and elderly group (aged 45 years and above). There were 16 people in the youth group and 18 people in the middle-aged and elderly group. The young group consisted of undergraduate students, whereas the senior group consisted of active or retired university employees. Due to the low popularity of smart homes among the elderly, the middle-aged group of 45–60 years is the generation of more intelligent technology, and they are also the future aging smart home use object, providing a reference value for smart technologies applications in the next 5–10 years. Informed consent was obtained from all participants.

The eye movement data of 4 participants were invalid, 1 from the young group and 3 from the senior group. These people’s eyes were swollen-lidded or their eyelids were pulled down due to cell aging, leading to a low sampling rate. Finally, 15 participants in the young group [7 male participants and 8 female participants, mean age = 24.2 years (SD = 1.8 years)] and 15 participants in the middle-aged and elderly group [8 male participants and 7 female participants, mean age = 63.0 years (SD = 6.1 years)] were included for data analysis (Table 1).

**TABLE 1:** Gender and age of subjects.

	Young group	Middle-aged and elderly group
**Age (years)**		
Range	[18,45)	[45,75)
Mean	24.2	63.0
SD	1.8	6.1
**Biologic sex**		
Female	8	7
Male	7	8

The average length and width of their index fingers were 72.3 mm (SD = 6.3 mm) and 14.3 mm (SD = 1.9 mm), respectively. The average whole-arm length was 61.7 cm (SD = 3.5 cm). All participants had a normal or corrected-to-normal vision, and none of them suffered color blindness. All the participants had experience in using touchscreen devices. In the youth group, 12 subjects had experience in using smart home devices, and 3 subjects did not. Only one of the subjects in the middle-aged and elderly group had experience in using smart home products, while the other 14 subjects did not.

### Materials and Tasks

**FIGURE 2:**
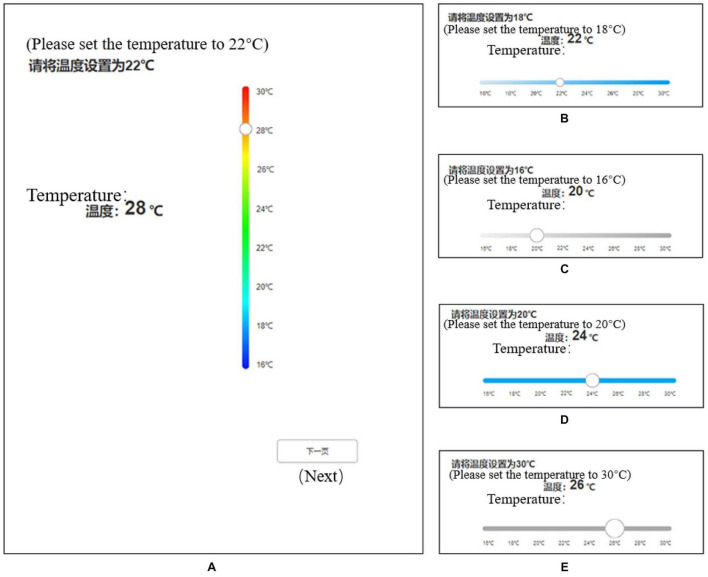
Examples of the screenshot for the experimental touchscreen interfaces. As shown in **(A)**, the experimental task is to adjust the temperature according to the text prompt at the top of the page by dragging the slider to the specified temperature. After completing the task on the page, the pilot presses the “Next” button to continue. **(A–E)** Present experimental material styles of different button sizes, track colors, and sliding orientation. English words in parentheses are used for explanation only and would not be shown in the test.

A smart home terminal interface prototype was developed with Axure and MockingBot. The prototype was presented on a Huawei tablet PC with EMUI 9.1 operating system (8.4 inches size with a resolution of 2,560 × 1,600 pixels). Referring to the previous study (Amrish et al., 2013; Chen et al., 2013), the display screen was at a 70° angle to the desk surface. Eye movements were sampled using an eye tracker (Tobii Pro Nano), with a sampling rate of 60 Hz and spatial accuracy of 0.3° or higher. The human-machine environment test cloud platform (ErgoLAB, Kingfar, China) was also put into use to measure the behavior data and eye-movement data. As shown in Figure 2, according to the dependent variables in the experiment, 30 sliding button forms of different factors and levels were made as experimental materials, and 3 groups of repeated measurement tasks were set for each form to calculate the average value and reduce the error, with a total of 90 experimental materials. Each page included a sliding component and a text prompt for the experimental task at the top, and the order of the 90 pages was scrambled. The background of the interface is white, and the text on the interface and buttons is black.

To eliminate the error due to different tasks, three groups of repeated measurement tasks are the same, but each of these same factors levels regulates the amount of temperature change task three times the sum of constant; for example, the slider with horizontal sliding direction, color gradient track color, and 3 mm button size, have three groups of different temperature settings: adjust from 22°C to 26°C, from 24°C to 20°C, from 22°C to 16°C. The temperature changes are 4, 4, and 6°C, respectively. The sum of the temperature changes of the other 29 sliding buttons for 3 adjustment tasks is also 14°C.

### Procedures

The experiment was conducted in the ergonomics laboratory of the Nanjing Forestry University. Before the experiment, participants were informed of the procedure of the experiment and they could stop at any time. After providing informed consent, participants were asked to fill out a personal information questionnaire including their demographic information and physical condition. A research assistant measured the length and width of the right index finger and the length of their right arm. Then, participants were tested for their cognitive ability by the Flanker task (Eriksen and Eriksen, 1974). After adjusting the seat to the appropriate angle and height and being informed about the operation procedure, participants were asked to complete the pretests and then the formal experiment began after the practice. The whole experiment was divided into three parts. Participants took a 3-min break after the completion of each part, and then, they continued with another part. Participants were required to complete the temperature setting task according to the text prompt as quickly and accurately as they could. Upon the completion of all parts, they were asked to fill out the user preference questionnaires about their button design preference. The whole experiment lasted for about 20 min.

### Data Analysis

First, to examine whether objective evaluation variables were normally distributed, the Shapiro-Wilk test was used, and three-way repeated-measures ANOVAs were used to analyze the effects of button size, track color, and sliding orientation. *Post-hoc* tests were performed using the Bonferroni correction for multiple comparisons. Sensitivity analyses were used to adjust the analyses for gender, the length and width of the index finger, and arm length in two groups but no significant effect was observed. A chi-square test was performed to examine the differences in user preference between the two groups. The significance level was set at *P* < 0.05 for all statistical tests. Statistical analyses were performed using IBM SPSS Version 22.

## Results

### Cognitive Ability

The flanker task was used to measure the cognitive ability of all participants. The variation coefficients of reaction time and accuracy rate of each one in the two groups were calculated, all of which were less than 0.15 (Table 2). It has been suggested that a coefficient of variation greater than 0.3 indicates that the data are faulty or that the experimental variables are uncontrollable (Brown, 1998). The results indicated that the dispersion of data was small, and the decision-making ability and the response ability of all participants were in a normal and equal range.

**TABLE 2:** Results of reaction time and accuracy rate on flanker tasks of all participants.

	Young group			Middle-aged and elderly group		
	Mean	SD	Coefficient of variation	Mean	SD	Coefficient of variation
Reaction time (ms)	501.249	59.896	0.119	677.822	101.16	0.149
Accuracy rate (%)	98.7	1.6	0.016	98.5	1.5	0.016

### Task Completion Time

Table 3 shows ANOVA results for task completion time. The sliding orientation and button size had a significant influence on the task completion time of the two age groups, while the track color had no significant influence on the task completion time of the young group, but had a significant influence on the middle-aged and elderly group. Both groups had shorter task completion time in the form of horizontal sliding, and the task completion time decreased with the increase in button size. Both groups had the shortest task completion time when the track color was color gradient, and the longest task completion time when the track color was blue gradient, but there was no significant difference between the young group and the middle-aged and elderly group.

**TABLE 3:** Main effects of sliding orientation, track color, and button size on task completion time (s).

	Young group				Middle-aged and elderly group			
	Descriptive analysis		ANOVA		Descriptive analysis		ANOVA	
	Mean	SD	*F*-values	*P*-values	Mean	SD	*F*-values	*P*-values
**Sliding orientation**								
Horizontal	3.775	0.926	25.826	< 0.001	6.436	2.554	21.997	< 0.001
Vertical	4.220	1.099			7.529	2.946		
**Track color**								
Color gradient	3.760	1.087	2.109	0.079	6.245	2.432	3.278	0.012
Blue gradient	4.144	0.936			7.483	2.960		
Gray gradient	4.043	0.987			7.230	2.894		
Blue solid color	4.018	1.140			6.840	2.830		
Gray solid color	4.022	1.018			7.111	2.792		
**Button size**								
3 mm	4.510	0.954	43.751	< 0.001	8.365	2.979	54.929	< 0.001
5 mm	3.976	1.058			7.187	2.635		
7 mm	3.507	0.848			5.396	1.853		

### Number of Saccades

Table 4 presents ANOVA results for a number of saccades in eye movement data. It was found that sliding orientation, track color, and button size had a significant influence on saccade times of the two age groups. In the sliding orientation factor, both groups had fewer saccades in horizontal sliding. In the track color factor, the two groups of subjects had the least number of saps in the color gradient slider; the young group had the most saps in the blue solid color slider, and the middle-aged and elderly group had the most saps in the gray solid color slider. In the button size factor, saccade times of both groups decreased with an increase in the button size.

**TABLE 4:** Main effects of sliding orientation, track color, and button size on number of saccades (times).

	Young group				Middle-aged and elderly group			
	Descriptive analysis		ANOVA		Descriptive analysis		ANOVA	
	Mean	SD	*F*-values	*P*-values	Mean	SD	*F*-values	*P*-values
**Sliding orientation**								
Horizontal	11.825	4.396	37.859	< 0.001	12.135	3.642	46.645	< 0.001
Vertical	15.222	8.267			14.187	5.280		
**Track color**								
Color gradient	11.685	5.414	4.838	0.001	11.337	3.202	14.049	< 0.001
Blue gradient	13.714	5.954			12.752	4.824		
Gray gradient	13.348	5.239			13.130	4.608		
Blue solid color	15.500	9.669			13.922	5.657		
Gray solid color	13.371	6.486			14.664	3.961		
**Button size**								
3 mm	17.004	7.720	45.947	< 0.001	16.922	5.374	157.376	< 0.001
5 mm	12.975	7.017			11.471	3.182		
7 mm	10.591	3.242			11.090	2.142		

**FIGURE 3:**
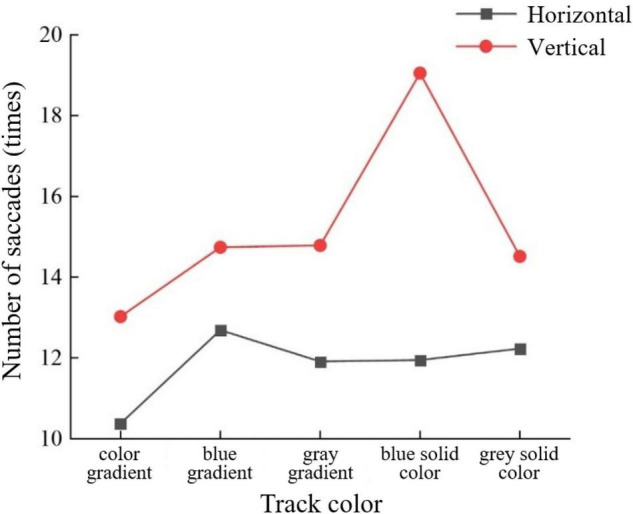
Number of saccades by track color and sliding orientation for the young group.

**FIGURE 4:**
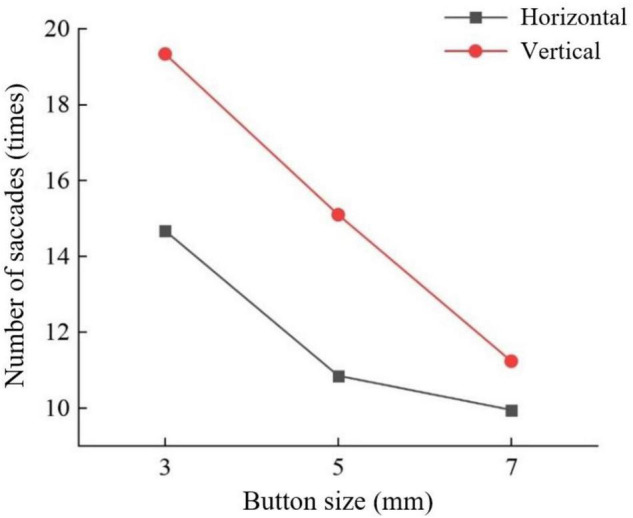
Number of saccades by button size and sliding orientation for the young group.

For the number of saccades in the young group, the slider track color had a significant interaction effect with the sliding orientation (*F* = 2.885, *P* < 0.05) (Figure 3), and the button size also had a significant interaction effect with the sliding orientation (*F* = 3.701, *P* < 0.05) (Figure 4). Horizontal sliding has fewer saccades regardless of button size and track color.

**FIGURE 5:**
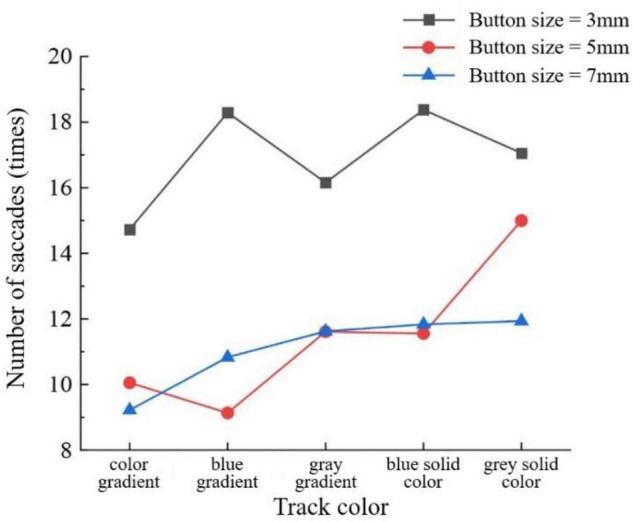
Number of saccades by track color and button size for the middle-aged and elderly group.

**FIGURE 6:**
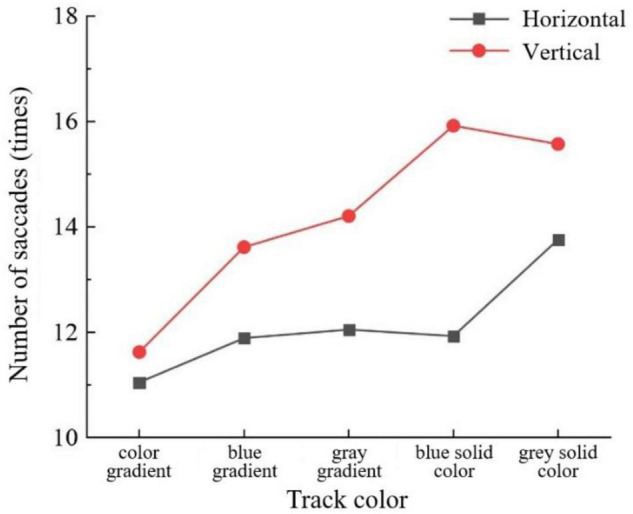
Number of saccades by track color and sliding orientation for the middle-aged and elderly group.

**FIGURE 7:**
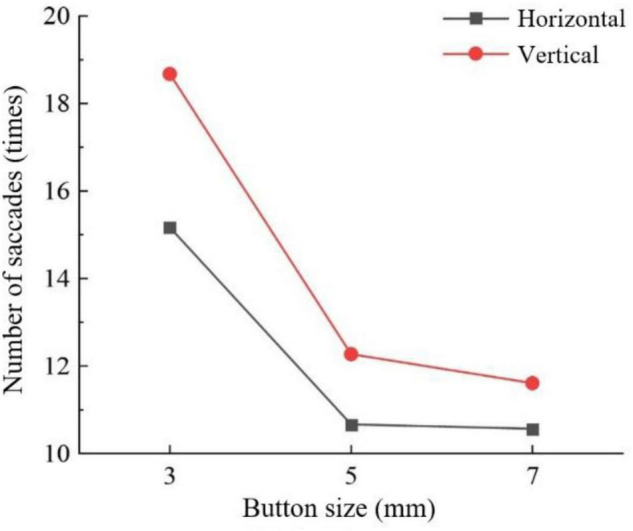
Number of saccades by button size and sliding orientation for the middle-aged and elderly group.

For saccades times of middle-aged and elderly group, track color interacted with button size (*F* = 5.705, *P* < 0.001) (Figure 5) and sliding orientation (*F* = 3.401, *P* < 0.05) (Figure 6). Button size interacted with sliding orientation (*F* = 6.168, *P* < 0.05) (Figure 7). The interaction effect of the three factors was significant. The 3-mm button size of all track colors has more number of saccades. In the two larger button sizes, the 5-mm button size of color gradient and gray pure color slider has more saccades times than the 7-mm button size, and the difference is more obvious under the latter condition. In the other three track colors, the 5-mm button size has less saccades times than the 7-mm button size and the difference is small. Horizontal sliding has fewer saccades regardless of button size and track color.

### Mean Fixation Time

Table 5 presents the results of ANOVA for mean fixation time in eye movement data. The sliding orientation and button size had a significant influence on the mean fixation time of the two age groups. The track color had no significant influence on the youth group but had a significant influence on the middle-aged and elderly group. For the sliding orientation factor, both groups had a shorter mean fixation time in the form of horizontal sliding. For the track color, both groups had a shorter mean fixation time in the form of color gradient, while the young group had a longer mean fixation time in the form of blue solid color, but there was no significant difference, while the middle-aged and elderly group had a longer mean fixation time in the form of gray gradient, and the difference was significant. The mean fixation time of the two groups decreased with the increase in button size.

**TABLE 5:** Main effects of sliding orientation, track color, and button size on mean fixation time (s).

	Young group				Middle-aged and elderly group			
	Descriptive analysis		ANOVA		Descriptive analysis		ANOVA	
	Mean	SD	*F*-values	*P*-values	Mean	SD	*F*-values	*P*-values
**Sliding orientation**								
Horizontal	0.348	0.090	12.684	< 0.001	0.353	0.106	6.122	0.014
Vertical	0.377	0.084			0.373	0.090		
**Track color**								
Color gradient	0.350	0.091	1.160	0.328	0.349	0.106	2.914	0.021
Blue gradient	0.366	0.091			0.374	0.103		
Gray gradient	0.355	0.084			0.384	0.104		
Blue solid color	0.371	0.082			0.352	0.092		
Gray solid color	0.370	0.091			0.357	0.084		
**Button size**								
3 mm	0.388	0.088	13.295	< 0.001	0.411	0.100	44.408	< 0.001
5 mm	0.361	0.087			0.359	0.091		
7 mm	0.338	0.083			0.320	0.081		

**FIGURE 8:**
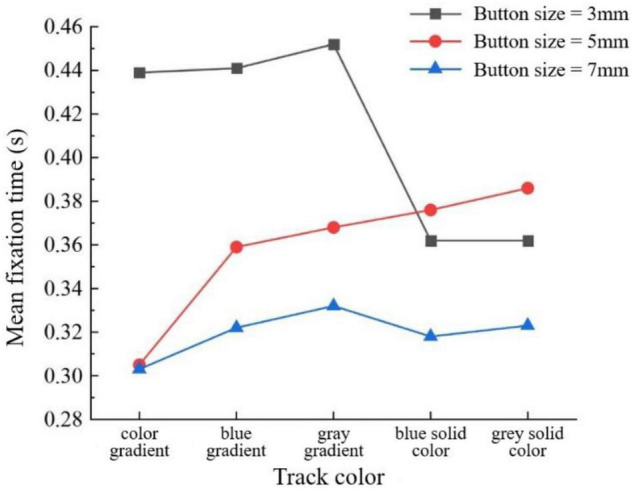
Mean fixation time (s) by track color and button size for the middle-aged and elderly group.

There was a significant interaction between track color and button size in the mean fixation time of the middle-aged and elderly group (*F* = 5.118, *P* < 0.001). As can be seen from Figure 8, among various track colors, the 7-mm button size has a shorter mean fixation time. For the gradient track, except that the 5- and 7-mm button size have the same mean fixation time with the color gradient track, the smaller the button size, the longer the mean fixation time. But in the pure color track, the 5-mm button size has a longer mean fixation time.

### User Preference

Table 6 shows user preference on button size, sliding orientation, and track color. In the young group, more people prefer horizontal sliding but the difference is not significant (60%; χ^2^ = 0.6, *P* > 0.05), prefer color gradient but the difference is not significant (60%; χ^2^ = 5.2, *P* > 0.05), and prefer 5-mm button size with a significant difference (73.3%; χ^2^ = 11.2, *P* < 0.05). None of the young group subjects preferred solid-color track. In the middle-aged and elderly group, there were significant differences in sliding orientation and track color. More of the middle-aged and elderly group subjects preferred horizontal sliding (93.3%; χ^2^ = 11.267, *P* = 0.001) and color gradient slider (86.7%; χ^2^ = 8.067, *P* = 0.005), and none of them preferred blue and gray gradient slider. In this group, more people prefer 7-mm sliders (53.3%; χ^2^ = 0.067, *P* > 0.05), but as many people prefer 5-mm sliders (46.7%), no one chooses 3-mm sliders.

**TABLE 6:** Distribution of user preference by sliding orientation, track color, and button size.

	Young group			Middle-aged and elderly group		
	Percentage	χ^2^	*P*-values	Percentage	χ^2^	*P*-values
**Sliding orientation**						
Horizontal	60.0%	0.6	0.607	93.3%	11.267	0.001
Vertical	40.0%			6.7%		
**Track color**						
Color gradient	60.0%	5.2	0.093	86.7%	8.067	0.007
Blue gradient	26.7%			0		
Gray gradient	13.3%			0		
Blue solid color	0			6.7%		
Gray solid color	0			6.7%		
**Button size**						
3 mm	6.7%	11.2	0.004	0	0.067	1
5 mm	73.3%			46.7%		
7 mm	20.0%			53.3%		

## Discussion

In this study, the performance of users in two age groups was experimentally studied by simulating the smart home terminal interaction interface for three interface design characteristics of the sliding component, namely, sliding orientation, track color, and button size. The experimental data includes behavioral indicators, eye movement indicators, and subjective indicators. The behavior indicator is the task completion time, that is, the total time it takes subjects to complete the specified task. The shorter the task completion time is, the better the performance of subjects is. Task completion time reflects the user performance in behavioral science. The eye movement index reflects the visual information processing and cognitive load of subjects. Saccade times and mean fixation time were selected as eye movement indicators in this experiment. Saccade number refers to the total number of the whole process from the beginning point to the end point of the behavior of the visual point moving quickly to another point. The more saccades times means the longer the search process and the greater the cognitive load. The mean fixation time is the mean of each time when the vision remains relatively static within a certain period, which is mainly used for the visual system to recognize and extract interface information. The longer the mean fixation time means the more difficult it is to extract information, the greater the cognitive load, or the more attractive the target is. The user subjective evaluation questionnaire is used to investigate the subjective preferences of the subjects.

Overall, the user performance of the young group was better than that of the middle-aged and elderly group. In the young group, the color of the slider had no significant influence, while the sliding orientation and button size had a significant influence. In the middle-aged and elderly group, all three factors have a significant influence on it, and there is an interaction among these three factors.

### Effects of Sliding Orientation

Different sliding orientations had an impact on the task completion time and eye movement indicators of the two age groups, and the participants had better performance with horizontal sliding. This is consistent with the research results of Colley (Colley et al., 2019), who believe that horizontal sliding is better than vertical sliding of touch screen interface, and horizontal sliding has smaller offset and higher accuracy. This study proves this from task completion time, saccade times, and mean fixation time. Both age groups have better user performance in the horizontal sliding direction.

The eye movement data of both groups showed the interaction between the sliding orientation and the other two factors. In different track colors and button sizes, the horizontal sliding direction always had less cognitive load than the vertical sliding direction. Therefore, when designing the sliding component on the interactive interface, the horizontal direction should be used as far as possible instead of the vertical direction.

### Effects of Track Color

The effect of track color on the two age groups is different. In the youth group, although the track color of color gradient had better operation performance, there was no significant difference between different colors, and the significant difference was only reflected in saccade times, while the effect on task completion time and mean fixation time was not significant. The middle-aged and elderly group have the best user performance and a significant impact on the slider with color gradient. Due to the degeneration of visual ability of the elderly, their vision is less sensitive to pure color or monochrome, and the slider with color gradient has a good reminder for them. The middle-aged and elderly group had the largest number of saccades in the gray solid color and the longest mean fixation time in the gray gradient. However, the interaction between the track color and the size of the slider showed that when the size of the slider increased to 7 mm, the user performance of the gray solid color slider also improved. The results of the study are not entirely consistent with our hypothesis. It can be said that the older the user, the stronger the guiding effect of color. Therefore, in the general design of track color, gradient color should be selected as far as possible, and strong color contrast can increase interface usability and user operation performance. However, in this study, the experimental task takes temperature control as an example, and red and blue with strong contrast can be used to represent high temperature and low temperature, respectively, which conforms to the daily cognition of users. Further research is still needed on how to choose the track color in other control tasks.

### Effects of Button Size

The button size had a significant influence on task completion time and eye movement index of the two age groups. As our research hypothesis, the user performance is improved with the increase in the size, and the two age groups have better performance in 5- and 7-mm button size. From the interaction between button size and sliding orientation, it can be seen that when the sliding orientation is horizontal, the button size of 5 mm will produce better performance, and the button size of 5 and 7 mm has less impact on the user performance. In the mean fixation time index of the middle-aged and elderly group, the interaction effect of track color and button size shows that, with the color gradient track, the user’s operation performance is poor at 3 mm, and the difference between 5 and 7 mm is not significant. If the area of the touch screen interface is limited, 5-mm slider is a more appropriate size. The size selection of this experiment is derived from the investigation of existing smart home interfaces, which is different from the size of the input button. Subsequent research can further explore the user performance of larger slider keys.

### User Preference

The results of the user subjective preference survey are basically consistent with the results of the user performance based on objective data. In terms of sliding direction, the proportion of horizontal sliding orientation was higher in both age groups: 60% in the young group and 93.3% in the middle-aged and elderly group. It has to be investigated further whether this large percentage difference is influenced by specific tasks. Young people have no obvious subjective preference for the sliding orientation. According to the interview, some people chose the vertical sliding direction because it is the same as the volume adjustment method of mobile phones, and it can reduce hand shielding, which is related to their personal operating habits. Or, to put it another way, they have developed a habit of using smartphones. The elderly also expressed that the horizontal sliding orientation is more consistent with the operation habits. According to the interview, many sliding control components used by them are horizontal sliding in real life. If the physical button is vertical sliding, it may be affected by aging and gravity problems. Furthermore, as compared to younger users, the elderly had a large preference difference in the horizontal and vertical orientations, which could be due to the fact that they are not affected by other smart devices such as mobile phones. The outcomes are more relevant to this research.

In terms of track color, people of both age groups prefer color gradient, and the preference of middle-aged and elderly group is more obvious. Young people said that they chose this form not because they like the colorful one, but because they think its recognition efficiency is better than other forms. A few young people think that this kind of color collocation is too flowery, and a blue gradient or a gray gradient track is already a good hint. No young people choose solid colors. Almost all the middle-aged and elderly adults chose the slider with a color gradient. They found the color attractive, and it served as a reminder that warm colors represented high temperatures and cool colors represented low temperatures. However, the generation of this preference is strongly linked to the experimental materials, and it has a substantial effect on users’ perceptions of context. It’s debatable if middle-aged and elderly users still prefer a red and blue gradient for slider track color in other contexts, or whether they prefer a single color of the gradient like the younger group in tasks such as volume adjustment or lighting brightness adjustment. The color of the blue gradient and gray gradient slider is partly light, which is difficult to be recognized by middle-aged and elderly people with degraded vision, so no middle-aged and elderly people choose monochromatic gradient. There are a small number of elderly people who choose blue and gray because of their personal preference.

As for the button size, the young group preferred 5 mm. Objective experimental data results show that the button size of 7 mm is better than 5 mm, but the young people said that the button size of 7 mm is too large, affecting the appearance, and the adjustment accuracy may decline. The middle-aged and elderly group preferred 7 mm, but the number of people who chose 5 mm was close to the number of people who chose 7 mm. This result supports the previous objective experimental data. Although 7 mm is better than 5 mm, there is no significant difference in user operation performance and subjective preference. The elderly think that the slider of 3 mm is too small and smaller than the width of fingers. In the case of a limited interface, the slider of 5 mm is a more appropriate size and has good operation performance.

## Conclusion

This study examined the influence of sliding orientation, track color, and button size of the sliding component in smart home display control interface on two age groups of young and middle-aged and elderly users. By simulating the temperature setting task of the system, questionnaire and interview, the results of objective indicators and subjective preferences were obtained. It was found that the users of the two age groups had the same performance for sliding orientation factors, while the results of track color and button size were different. The user performance of the youth group was better in horizontal sliding direction and button size of 5 mm and above, with a preference for button size of 5 mm, while track color had less influence on user performance. The user performance of the middle-aged and elderly group is better in horizontal sliding direction, color gradient track, and button size of 5 mm and above. This experiment aims to understand the influence of smart home interface button features on different age groups. Designers and engineers can give priority to the middle-aged and elderly group in product design and development, expand user groups on the premise of meeting the requirements of the elderly, and improve product versatility. However, since the subjects were divided into two groups, namely, young group and middle-aged and elderly group; each group’s age range was wide, and the results of this study could only indicate differences in user performance due to a large age gap. Subsequent research can further subdivide the subjects’ ages.

## Data Availability Statement

The raw data supporting the conclusions of this article will be made available by the authors, without undue reservation.

## Ethics Statement

Ethical review and approval was not required for the study on human participants in accordance with the local legislation and institutional requirements. The patients/participants provided their written informed consent to participate in this study.

## Author Contributions

NY contributed to the idea of this study, reviewed the draft, and made revisions. ZO assisted in the experiment and wrote most of the manuscript. HW contributed to the participants’ recruitment and experimental design. All authors contributed to the article and approved the submitted version.

## Conflict of Interest

The authors declare that the research was conducted in the absence of any commercial or financial relationships that could be construed as a potential conflict of interest.

## Publisher’s Note

All claims expressed in this article are solely those of the authors and do not necessarily represent those of their affiliated organizations, or those of the publisher, the editors and the reviewers. Any product that may be evaluated in this article, or claim that may be made by its manufacturer, is not guaranteed or endorsed by the publisher.

## Appendix

### Appendix A: Preference Questionnaire

**Table:**

		Subject No.________
Please choose an option that best describes your answer for each question.		
1. Which button size do you prefer most?		

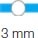
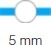
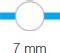
		
2. Which color of the track do you prefer most?		

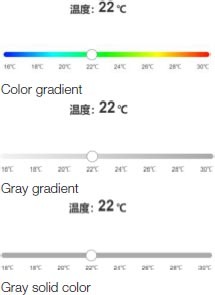
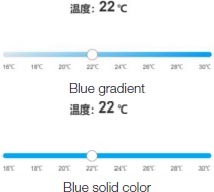
		
3. Which sliding orientation do you prefer most?		
		
